# Differential effects of ibuprofen and indomethacin on cerebral oxygen kinetics in the very preterm baby

**DOI:** 10.3389/fped.2022.979112

**Published:** 2022-10-03

**Authors:** Michael J. Stark, Tara M. Crawford, Nina M. Ziegler, Anthea Hall, Chad C. Andersen

**Affiliations:** ^1^Department of Neonatal Medicine, The Women’s and Children’s Hospital, North Adelaide, SA, Australia; ^2^Robinson Research Institute, School of Medicine, The University of Adelaide, Adelaide, SA, Australia

**Keywords:** preterm neonate, patent ductus arterious, indomethacin, ibuprofen, oxygen delivery, oxygen consumption, fractional oxygen concentration

## Abstract

**Background:**

Ibuprofen is preferred to indomethacin for treatment of a significant patent ductus arteriosus (PDA) in preterm babies despite indomethacin being associated with a lower risk of intraventricular haemorrhage. This difference is thought to relate to the discrepant effects of each medication on cerebral oxygen kinetics yet the effect of ibuprofen on cerebral perfusion is uncertain.

**Methods:**

Forty-eight babies < 30 weeks with a significant PDA, defined by echocardiography, were randomly assigned to either indomethacin or ibuprofen (*n* = 24 per group) and stratified by gestation and chronologic age. Cerebral blood flow [total internal carotid blood flow (TICF)] and oxygen physiology [oxygen delivery (modCerbDO_2_) and consumption (modCerbVO_2_)] were measured using cranial Doppler ultrasound and near-infrared spectroscopy, and cerebral oxygen extraction (cFTOE) calculated, immediately before and following administration. Temporal and treatment related changes were analysed.

**Results:**

A fixed effect of time was seen for TICF (*p* = 0.03) and therefore modCerbDO_2_ (*p* = 0.046) and cFTOE (*p* = 0.04) for indomethacin alone. In the indomethacin group, TICF and modCerbDO_2_ fell from baseline to 5 and 30 min respectively (TICF *p* < 0.01, cDO_2_ *p* = 0.01) before increasing from 5 min to 24 h (*p* < 0.01) and 30 min and 24 h (*p* < 0.01) timepoints. cFTOE peaked at 30 min (*p* = 0.02) returning to baseline at 24 h. There was a parallel increase in arterial lactate.

**Conclusion:**

Indomethacin significantly reduces cerebral blood flow soon after administration, resulting in a parallel increase in oxygen extraction and arterial lactate. This implies that the balance of oxygen kinetics at the time of treatment may be critical in very preterm babies with significant PDA.

## Introduction

Management of a patent ductus arteriosus (PDA) in preterm babies remains contentious with treatment approaches varying between centres and individual clinicians. The Cochrane systematic review of the comparative effectiveness of the two commonly used NSAIDs, indomethacin and ibuprofen, concludes that both medications are equally efficacious when used for ductal closure (1) though ibuprofen results in fewer side effects. Unfortunately however, neither medication appears to advantageously alter long term outcome (2, 3). Moreover, there is recent trial evidence showing that a conservative compared with an active management approach to ductal closure does not adversely alter either mortality or morbidity, creating further clinical uncertainty (4).

Notwithstanding the overall finding of the Cochrane review, there are further knowledge gaps. In particular, the precise definition of a *clinically significant patent ductus*, in addition to the timing, dosing pattern and most appropriate agent for ductal closure in preterm babies (5). More tantalising though is the finding of reduced severe intraventricular haemorrhage (IVH) with use of indomethacin, an effect not seen with ibuprofen (3, 6, 7). This outcome, originally reported by Ment et al., and subsequently confirmed in the Trial of Indomethacin Prophylaxis in Preterms (TIPP), has never been adequately explained though both these trials focus primarily on prophylaxis rather than treatment and include cohorts from a different era with lower use of antenatal steroids (6, 7).

It has been proposed that the potential *protective* effect of indomethacin relates to either reduced cerebral perfusion or cerebral blood flow fluctuation or both in combination (8–10). Although oxygen kinetics vary in each newborn the fundamental principles of oxygen physiology still apply, making it hard to reconcile the enticing effect of indomethacin without further information about contemporaneous cerebral oxygen kinetics (11). It was the aim of the current study to determine the temporal changes in cerebral oxygen kinetics in very preterm babies with a significant PDA following administration of either indomethacin or ibuprofen.

## Materials and methods

**FIGURE 1:**
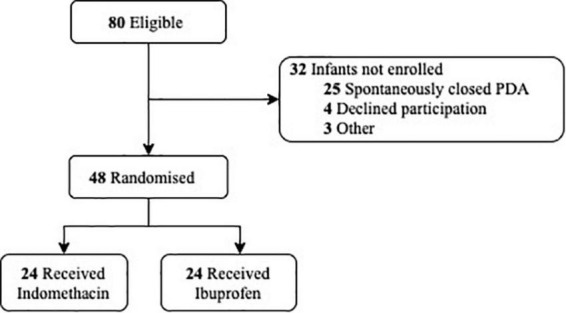
Study flow diagram.

We conducted a randomised trial of indomethacin compared with ibuprofen for targetted PDA treatment in the neonatal intensive care unit of The Women’s and Children’s Hospital, Adelaide, South Australia from April 2019 to March 2020. Preterm newborns born at <30 weeks’ gestation with a significant PDA confirmed by echocardiography, were eligible for recruitment (12). Those with life-threatening congenital abnormalities or congenital heart disease were excluded. A computer-generated randomisation schedule using variable block sizes were generated and stratified by gestation (<25 and >25 weeks) and postnatal age at time of treatment (<12 and >12 h) with a 1:1 allocation ratio. Written informed consent was obtained prior to treatment (Figure 1). The Women’s and Children’s Human Research Ethics Committee approved the study protocol (HREC/16/WCHN/175).

Enrolled newborns were nursed in a neutral thermal range. The nursery uses a cutaneous oximetry target of (90–95%) and pCO_2_ (45–55 mmHg) in babies requiring supplemental oxygen and/or respiratory support and has a standardised approach to use of inotropes. Measurements were obtained with the incubator in a horizontal position and the infant settled in supine, immediately prior to the first dose of either indomethacin or ibuprofen and subsequently at 5 min, 30 min, 4 h, and 24 h after completion of the drug infusion. The ibuprofen group received a 20 mg/kg dose by intravenous infusion over 15 min (Pedea, Recordati Rare Disease Australia, Sydney, Australia), while the indomethacin group received 0.2 mg/kg administered by intravenous infusion over 30 min (Ductaclose, Samarth Life Sciences, Mumbai, India). Only the response to the first dose of either treatment was studied.

### Cerebral oxygen delivery and consumption

The tissue oxygen index (TOI) was measured by near-infrared spectroscopy (NIRS) (NIRO-200NX; Hamamatsu Photonics, Hamamatsu City, Japan). The sensor was placed on the right frontotemporal region with data captured at 1-s intervals. A continuous period was recorded from the pre-treatment timepoint until 4 h after completion of the drug infusion. A further 30-min continuous period was recorded at 24 h. Five-minute epochs of stable TOI data at each time point were averaged and used in the oxygen kinetic equations as a surrogate for cerebral venous oxygen saturation.

Serial pulsed-wave Doppler ultrasound measurement of the internal carotid artery was performed by a single operator (AH) using an 8-MHz linear phased-array transducer (Philips iE33 Ultrasound System; Philips Healthcare, Andover, MA, United States) according to previously published method (13).

### Other measurements

An arterial blood sample was obtained and analysed (ABL 725 spectrophotometer; Radiometer, Copenhagen, Denmark) with each NIRS measurement. Heart rate, invasive mean blood pressure, inspired oxygen concentration, mean airway pressure, temperature, and concurrent therapies were recorded.

### Calculations

Modified cerebral oxygen delivery (mCerbDO_2_, mL/kg/min) was calculated using the formula mCerbDO_2_ = [CBF × ((1.39 × Hb × Hbsat/100) + (0.003 × PaO_2_))], where TICF (mL/kg/min) is a surrogate for CBF, Hb is Hb concentration (g/dL), and Hbsat is Hb saturation (%). Modified cerebral oxygen consumption (mCerbVO_2_, mL/kg/min) was calculated according to the Fick principle (11). cFTOE (%) was calculated using the formula cFTOE = [(SaO_2_-TOI)/SaO_2_], where SaO_2_ is co-oximetry–derived arterial oxygen saturation and TOI used in place of cerebral venous oxygen saturation.

### Statistical analysis

Sample size was calculated from published data regarding the observed change in cFTOE 30 min following treatment with indomethacin (11). Based on this data, a sample size of 21 newborns per group was needed to provide 90% power to detect a 1SD difference in the 30-min cFTOE between the 2 treatment groups with an α = 0.05.

Differences between the groups were assessed using independent-samples *t* tests for continuous variables displaying normal distribution and the Wilcoxon rank-sum test for variables not normally distributed. Differences in frequencies for categorical variables were tested using the χ^2^ test or Fisher exact test. Within subject temporal changes and rate of change between study time-points in cerebral blood flow, oxygen delivery, oxygen consumption and cFTOE were analysed using Mixed Linear Models with treatment group the fixed factor. Heterogenous compound symmetry was used as the repeated covariance type and Fisher Unprotected Least Significant Difference (LSD) as the confidence interval adjustment to compare changes within treatment groups. To control for repeated comparisons, *p* = 0.005 was used to determine significance for *post hoc* analysis of differences between study time-points time points and *p* = 0.008 for differences in the rate of change for the oxygen kinetic variables. Data were analysed using the Statistical Package for the Social Sciences (SPSS v28; IBM SPSS, Chicago, IL, United States). The study statistician was blinded to allocated therapy.

## Results

The clinical characteristics of the 48 newborns are shown in Table 1. While newborns in the ibuprofen group had higher birthweights, the rate of SGA between the groups was similar. PDA size and postnatal age at the time of treatment did not differ. There was no significant difference in the need for ventilatory or haemodynamic support between groups, with dobutamine the only inotrope administered. In addition, there were no differences in cerebral oxygen kinetics at baseline.

**TABLE 1:** Clinical characteristics.

	Indomethacin (*n* = 24)	Ibuprofen (*n* = 24)	*P*
Gestation (days)	186 (179–194)	192 (179–200)	0.19
Birth weight (kg)	920 (793–1118)	1038 (741–1285)	0.01
Sex (male)	13 (54%)	10 (40%)	0.1
Histological chorioamnionitis	12 (50%)	11 (46%)	0.5
SVD	12 (50%)	9 (38%)	0.26
SGA	4 (17%)	6 (25%)	0.35
Completed course of antenatal steroids	20 (83%)	18 (75%)	0.4
Mechanically ventilated	14 (58%)	15 (63%)	0.37
Inotropes	12 (50%)	13 (54%)	0.38
PDA Size (mm)	2.1 (1.8–3.0)	2.3 (1.6–2.5)	0.36
Age at treatment (h)	16 (8–24)	19 (10–33)	0.09
Ductal closure after single dose	14 (58%)	15 (62.5%)	0.5
IVH	5 (21%)	6 (25)	0.5

Data presented as median (IQR) or *N* (%). Kruskal–Wallis test and Pearson Chi-Square used for comparison between groups. SVD, spontaneous vaginal delivery; SGA, small for gestational age; PDA, patent ductus arteriosus; IVH, intraventricular haemorrhage (any grade). Ductal closure was determined by clinical assessment and examination.

### Total internal carotid flow

**FIGURE 2:**
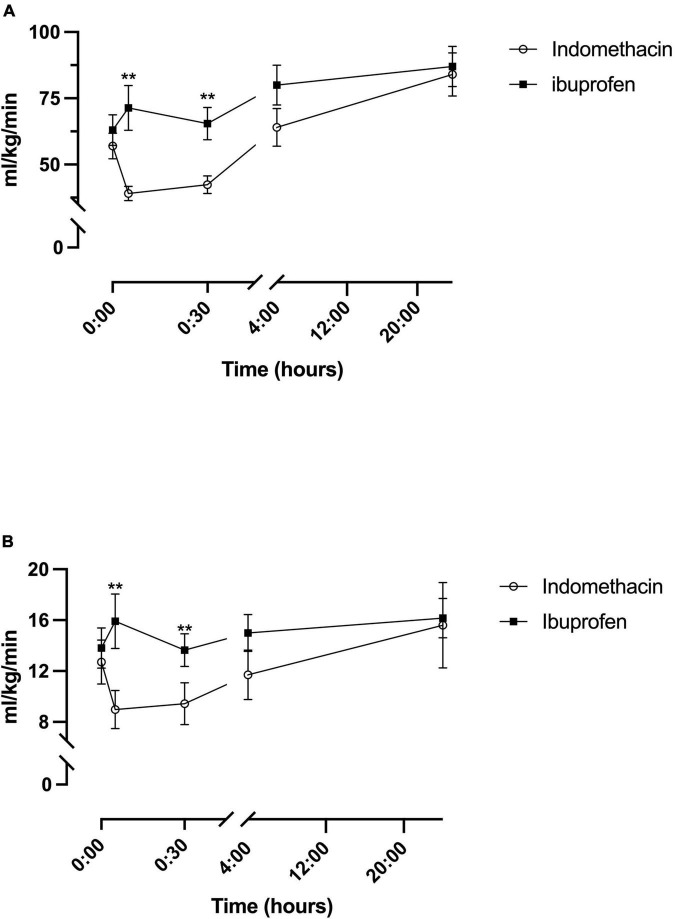
Temporal changes in oxygen delivery for the indomethacin and ibuprofen treatment groups. **(A)** Total internal carotid blood flow. **(B)** modCerbDO_2_. **p < 0.01 between treatment group difference at that study timepoint.

A fixed effect for time was seen for indomethacin (*p* < 0.001) and ibuprofen (*p* = 0.02) (Table 2). For the indomethacin group, TICF fell between baseline and the 5 (*p* < 0.001) and 30-min (*p* = 0.001) timepoints before increasing above baseline levels from the 4-h timepoint onwards, baseline to 24 h (*p* = 0.002) 5-min to 4 h (*p* = 0.003), 5-min to 24 h (*p* < 0.001); and 30 min to 24 h (*p* < 0.001) (Figure 2A). For babies in the ibuprofen group, the only significant change in flow occurred between baseline and 24 h (*p* = 0.003). Mixed-model analysis also showed a fixed effect for treatment (*F*_(1–30_._5)_ = 6.3, *p* = 0.017). On average, TICF was 19.8 (95% CI, -35.8 to -3.7) ml/kg/min lower in the indomethacin group. *Post hoc* analysis demonstrated differences between the groups at the 5 min (*p* = 0.002) and 30-min time-points (*p* = 0.005).

**TABLE 2:** Mixed linear models fixed effect for time for the measured oxygen kinetic variables by indomethacin and ibuprofen treatment group.

Oxygen kinetics variable		Time effect	*P*
TICF	Indomethacin	*F*_(1–67_._9)_ = 16.5	<0.001
	Ibuprofen	*F*_(1–51_._8)_ = 5.4	0.02
mCerbDO_2_	Indomethacin	*F*_(1–80_._8)_ = 6.1	0.016
	Ibuprofen	*F*_(1–55_._5)_ = 0.13	0.91
mCerbVO_2_	Indomethacin	*F*_(1–63_._7)_ = 7.4	0.008
	Ibuprofen	*F*_(1–62_._5)_ = 0.44	0.5
FOE	Indomethacin	*F*_(1–93_._4)_ = 5.8	<0.001
	Ibuprofen	*F*_(1–69_._4)_ = 0.31	0.58
Lactate	Indomethacin	*F*_(1–56_._7)_ = 3.52	0.04
	Ibuprofen	*F*_(1–48_._9)_ = 0.12	0.7

### Modified cerebral oxygen delivery

As a result of the changes to TICF above, there was a fixed effect for time seen within the indomethacin group (*p* = 0.016) (Table 2). *Post hoc* analysis demonstrated an increase in mCerbDO_2_ between the 5 min and 24-h timepoint (*p* < 0.001) and the 30 min and 24 h timepoints (*p* = 0.001) (Figure 2B). As with TICF, there was a fixed effect for treatment (*F*_(1–32_._8)_ = 6.5, *p* = 0.016) (Figure 2B). mCerbDO_2_ was on average 4.5 (95% CI, -9.1 to -1.0) ml/kg/min lower in the indomethacin group. *Post hoc* analysis demonstrated differences between the at the 5 min (*p* = 0.007) and 30-min timepoints (*p* = 0.005).

### Modified cerebral oxygen consumption

**FIGURE 3:**
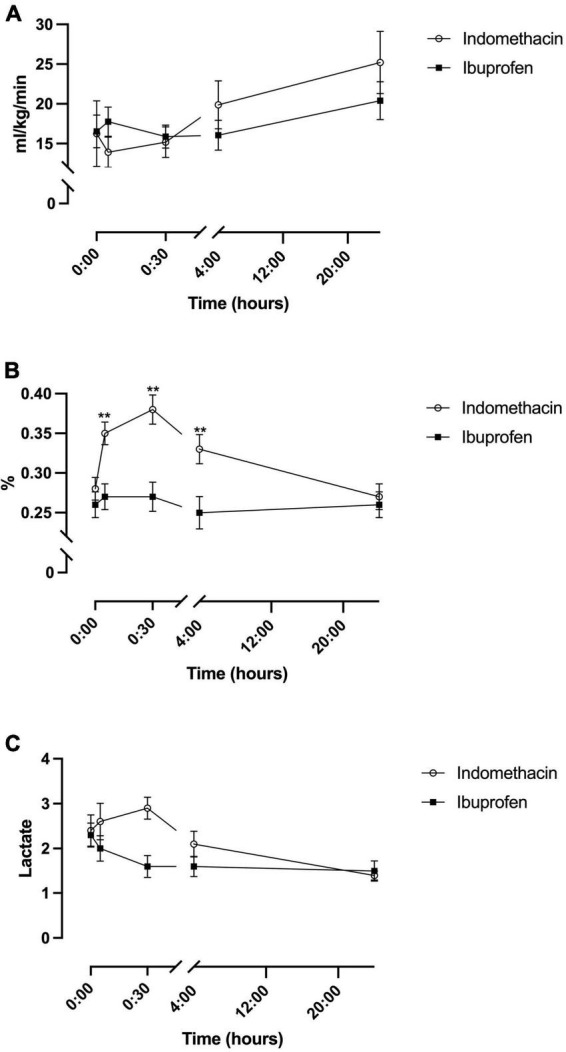
Temporal changes in oxygen consumption for the indomethacin and ibuprofen treatment groups. **(A)** modCerbVO_2_. **(B)** Cerebral fractional oxygen extraction. **(C)** Arterial lactate. **p < 0.01 between treatment group difference at that study timepoint.

A fixed effect for time was seen for the indomethacin group (*p* = 0.008), but not the ibuprofen group (Table 2). *Post hoc* analysis demonstrated an increase in mCerbVO_2_ between the 5 min and 24-h timepoint (*p* = 0.002) and the 30 min and 24 h timepoints (*p* = 0.004). No fixed effect for treatment group was seen (Figure 3A).

### Cerebral fractional oxygen extraction and lactate

For cFTOE a fixed effect for time and treatment was seen (*F*_(1–52_._9)_ = 4.1, *p* = 0.045). cFTOE did not significantly change in the ibuprofen group whereas, in the indomethacin group, cFTOE increased in the immediate post-treatment period peaking at 30 min (Figure 3B). *Post hoc* analysis demonstrated that this increase was significant between the baseline and 5-min (*p* < 0.001), baseline and 30-min (*p* < 0.001), and 5-min and 30-min (*p* = 0.002) timepoints. cFTOE then returned to baseline levels with differences in extraction seen between the 5-min and 24-h (*p* = 0.001) and 30-min and 24-h timepoints (*p* = 0.001). On average cFTOE was 0.07 (95% CI, 0.02 to 0.12) higher in the indomethacin group. *Post hoc* analysis demonstrated differences between the groups at the 5-min (*p* = 0.002), 30-min (*p* = 0.001), and 4-h time-points (*p* = 0.004) (Figure 3B).

For arterial lactate, there was a fixed effect of time in the indomethacin group (*p* = 0.04). *Post hoc* analysis demonstrated an increase in lactate from baseline to 30 min that neared significance (*p* = 0.02). Lactate decreased between the 5 min and 24-h (*p* < 0.001), 30 min and 4-h (*p* = 0.004), 30 min and 24-h (*p* < 0.001) and 4 h and 24-h (*p* = 0.003) timepoints. No fixed effect for treatment group was seen (Figure 3C).

### Rate of change following treatment administration

Mixed linear models were used to determine if the rate of change in TICF and the NIRS derived measures of cerebral oxygen delivery and consumption between the time points was different between the treatment groups. The Δchange for the 4-time epochs (Epoch 1: baseline to 5 min; Epoch 2: 5–30 min; Epoch 3: 30 min to 4 h and Epoch 4: 4–24 h) was calculated and divided by the duration of the epoch in minutes.

A fixed effect for treatment group was seen for ΔTICF (*F*_(1–156)_ = 8.4, *p* = 0.004). *Post hoc* analysis demonstrated that the ΔTICF was significantly different for Epoch 1 (*p* = 0.001). No significant fixed effect for treatment group was observed for ΔDO_2_, ΔVO_2_ or lactate. Finally, a fixed effect for treatment group was seen for ΔcFTOE (*F*_(1–154)_ = 8.8, *p* = 0.003) with significant differences in ΔcFTOE in Epochs 1 (*p* = 0.001) and 4 (*p* = 0.008).

## Discussion

The kinetics of cerebral oxygen metabolism is particularly dynamic in the first few days following preterm birth. Added to this is the effect of vasoactive medication prescribed for the treatment of the clinically significant PDA. This makes for a biologically noisy environment in the very preterm baby. This study measured the effect of either indomethacin or ibuprofen, given according to standard dosing patterns, on cerebral oxygen kinetics in a group of very preterm babies with a PDA defined according to a previous randomised trial (14). Indomethacin in comparison to ibuprofen, significantly reduces cerebral blood flow and therefore, cerebral oxygen delivery, with a nadir shortly after administration. Despite this, cerebral oxygen consumption was similar between the groups as a result of an increase in cFTOE in the indomethacin group, with peak extraction approaching a value previously reported to be associated with poor outcome. The implication of a high peak cFTOE value in very preterm newborns assigned to indomethacin, is highlighted by a simultaneous increase in systemic lactate suggesting that this approach may imperil the dynamic balance between oxygen consumption and delivery in very preterm babies (11). Given the comparable rate of ductal closure, the potential for differential effects of indomethacin and ibuprofen on cerebral oxygen physiology is significant and should therefore be carefully considered by the treating clinician (9).

### Kinetics of oxygen metabolism

For the extremely preterm newborn the early postnatal period is typically characterised by low baseline cerebral blood flow, high oxygen demand, and elevated cerebral oxygen extraction (11). Low cerebral artery blood flow, during transition, is associated with adverse neurologic outcome at age 12 months (15), while fluctuating flow increases the risk of IVH (16). In keeping with previously published data in very preterm newborns without evidence of IVH (11) on cranial sonagraph there is a dramatic fall in cerebral blood flow immediately following indomethacin administration (9, 17, 18). However, recovery of cerebral blood flow to baseline levels is slower in the current study than previously described, likely reflecting differences in methods, timing, and frequency of measurement.

As oxygen delivery falls or oxygen consumption increases, or both occur in combination, the amount of oxygen extracted from Hb increases thereby reducing the amount left over in the venous oxygen compartment (19). The response of extraction to the observed alterations in cerebral oxygen physiology is the most striking finding, providing a clear difference between the effect of indomethacin and ibuprofen on cerebral oxygen physiology. It is no surprise that the changes in cerebral blood flow from indomethacin administration provoke a response from oxygen extraction as this is the primary method of compensation for low oxygen delivery. cFTOE is already increased in newborns with a significant PDA, and while the underlying mechanism is unclear, it is thought to reflect a cerebral steal phenomenon, resulting in a reduction in cerebral blood flow during the cardiac cycle (20).

Oxygen extraction is a useful surrogate marker of imbalance to oxygen kinetics. cFTOE increases in the indomethacin group immediately following treatment, peaking at 30 min but remaining higher in comparison with ibuprofen across the duration of the study, a finding consistent with previously published animal (21, 22) and human data (18). Theoretically, oxygen consumption is restricted by oxygen delivery when overall compensation is exhausted resulting in anaerobic respiration and accumulation of lactate (11). In this study, the finding of an increase in arterial lactate in the indomethacin group, temporally related to the change in cFTOE, suggests that the relationship between oxygen consumption and delivery is unbalanced and approaching a critical threshold. It is possible that this finding may also reflect the vasoconstrictive effect of indomethacin on vascular beds other than the brain given the measure is systemic not cerebral (8). Animal models have shown that the critical venous oxygen threshold at which anaerobic metabolism develops is dependent on aetiology being lowest in hypoxic hypoxia and highest in stagnant hypoxia highlighting the importance of flow to compensation (23). It is unclear if a similar pattern is observed in preterm babies in whom a mixed model of hypoxaemia is most typical. Notwithstanding the important changes to cerebral oxygen kinetics from administration of indomethacin in particular, the risk of tissue injury is unknown with likely differing injury thresholds for mild but prolonged, brief but severe, and repetitive insults (11).

### Dosing and pharmacologic action of ibuprofen and indomethacin

The difference in administration of ibuprofen and indomethacin may explain some of the study findings. In contrast, when indomethacin is given by continuous infusion there is elimination of the fall in cerebral, renal and mesenteric blood flows (24, 25). There are also differences in pharmacologic action which may impact both systemic and cerebral vascular responses. Indomethacin is known to inhibit both isoforms of cyclo-oxygenase inhibitor (COX 1 & 2), though with greater selectivity for COX- 1 compared with COX-2 (26), resulting in greater systemic, non-selective overall vasoconstriction (27). In contrast, ibuprofen is a more potent inhibitor of COX-2 (28). This differential enzyme effect. In acute intermittent hypoxia, non-selective COX inhibition increases mean arterial blood pressure without effects on cerebral blood flow while selective COX-2 inhibition abrogates the intermittent hypoxia-induced increase in blood pressure and results in altered cerebral blood flow (29).

### Study limitations

There are recognised limitations in measurement of blood flow by Doppler ultrasound, oxygen consumption by the Fick and use of NIRS as a surrogate for cerebral venous oxygen saturation. Despite advances in NIRS techniques, the fixed arteriovenous partition ratio, output variability and lack of quantification continue to be barriers to clinical application (30). In this study, NIRS data was acquired from a single sensor placed on the right fronto-temporal region in a distribution supplied by the carotid circulation. Even so, in newborns without intraventricular haemorrhage, there is minimal variation between regions of the brain (31). In addition, episodic sampling of dynamic measures is also subject to over interpretation, particularly given the inherent biologic variability. While the measurement intervals are short, they are in keeping with similar measures of other variables in this population and are taken in similar environmental conditions. As this was a pragmatic randomised trial in a clinical setting, we did not perform serial echocardiography at the study timepoints to formally assess the duct following treatment, nor is it local practice to echocardiographically determine “closure” as definition of ductal closure remains indistinct. This makes it difficult to definitively determine if our findings are the result of the treatments themselves or the ductal effect of treatment. However, a comparable proportion of newborns in each group only required a single dose to achieve clinically defined ductal closure. Finally, it is important to acknowledge that not every study has reported adverse effects on cerebral oxygen delivery in the setting of a hemodynamically significant PDA (32) with cerebral autoregulation proposed to be important. The current study can make no finding of the effect of either medicine on cerebral autoregulation as this would require a different methodological approach.

### Summary

Indomethacin, given by bolus injection to very preterm babies with a clinically significant patent ductus arteriosus, significantly reduces TICF. As a result, cerebral oxygen delivery falls and oxygen extraction increases to maintain cerebral oxygen consumption. This alteration to cerebral oxygen kinetics was not seen with ibuprofen given by slow infusion. In this study, very preterm babies receiving indomethacin have an increase in systemic lactate coinciding with a significant reduction in oxygen delivery. This suggests that there is an adverse oxygen environment and cautions against using indomethacin in this population using the dosing pattern set out in this study.

## Data availability statement

The raw data supporting the conclusions of this article will be made available by the authors, without undue reservation.

## Ethics statement

The studies involving human participants were reviewed and approved by Women’s and Children’s Health Network Human Ethics Committee. Written informed consent to participate in this study was provided by the participants’ legal guardian/next of kin.

## Author contributions

MS, NZ, and CA conceptualised the study. TC and AH recruited subjects and performed all study investigations. MS analysed the data and prepared the manuscript. CA critically reviewed the manuscript. All authors contributed to the article and approved the submitted version.

## Abbreviations

PDA: patent ductus arteriosus
IVH: intraventricular haemorrhage
TICF: total internal carotid blood flow
modCerbDO_2_: cerebral oxygen delivery
modCerbVO_2_: cerebral oxygen consumption
cFTOE: cerebral fractional oxygen extraction
CMRO_2_: cerebral metabolic rate of oxygen
NIRS: near infra-red spectroscopy
TOI: tissue oxygenation index
COX: cyclo-oxygenase.

## References

[1] Ohlsson A Walia R Shah SS. Ibuprofen for the treatment of patent ductus arteriosus in preterm and/or low birth weight infants. *Cochrane Database Syst Rev.* (2013) 2013:CD003481. 10.1002/14651858.CD003481.pub5 23633310

[2] Fowlie PW Davis PG McGuire W. Prophylactic intravenous indomethacin for preventing mortality and morbidity in preterm infants. *Cochrane Database Syst Rev.* (2010) 2010:CD000174. 10.1002/14651858.CD000174.pub2 20614421 PMC7045285

[3] Ohlsson A Shah SS. Ibuprofen for the prevention of patent ductus arteriosus in preterm and/or low birth weight infants. *Cochrane Database Syst Rev.* (2020) 1:CD004213. 10.1002/14651858.CD010061.pub4 31985838 PMC6984616

[4] Letshwiti JB Semberova J Pichova K Dempsey EM Franklin OM Miletin J. A conservative treatment of patent ductus arteriosus in very low birth weight infants. *Early Hum Dev.* (2017) 104:45–9. 10.1016/j.earlhumdev.2016.12.008 28042972

[5] Parkerson S Philip R Talati A Sathanandam S. Management of patent ductus arteriosus in premature infants in 2020. *Front Pediatr.* (2020) 8:590578. 10.3389/fped.2020.590578 33643964 PMC7904697

[6] Ment LR Oh W Ehrenkranz RA Philip AG Vohr B Allan W Low-dose indomethacin and prevention of intraventricular hemorrhage: a multicenter randomized trial. *Pediatrics.* (1994) 93:543–50. 10.1542/peds.93.4.543 8134206

[7] Schmidt B Davis P Moddemann D Ohlsson A Roberts RS Saigal S Long-term effects of indomethacin prophylaxis in extremely-low-birth-weight infants. *N Engl J Med.* (2001) 344:1966–72. 10.1056/NEJM200106283442602 11430325

[8] Yanowitz TD Yao AC Werner JC Pettigrew KD Oh W Stonestreet BS. Effects of prophylactic low-dose indomethacin on hemodynamics in very low birth weight infants. *J Pediatr.* (1998) 132:28–34. 10.1016/S0022-3476(98)70480-9 9469996

[9] Patel J Roberts I Azzopardi D Hamilton P Edwards AD. Randomized double-blind controlled trial comparing the effects of ibuprofen with indomethacin on cerebral hemodynamics in preterm infants with patent ductus arteriosus. *Pediatr Res.* (2000) 47:36–42. 10.1203/00006450-200001000-00009 10625080

[10] Andersen CC Hodyl NA Kirpalani HM Stark MJ. A theoretical and practical approach to defining “adequate oxygenation” in the preterm newborn. *Pediatrics.* (2017) 139:e20161117. 10.1542/peds.2016-1117 28325811

[11] Balegar KK Stark MJ Briggs N Andersen CC. Early cerebral oxygen extraction and the risk of death or sonographic brain injury in very preterm infants. *J Pediatr.* (2014) 164:475.e–80.e. 10.1016/j.jpeds.2013.10.041 24360993

[12] Shepherd JL Noori S. What is a hemodynamically significant PDA in preterm infants? *Congenit Heart Dis.* (2019) 14:21–6. 10.1111/chd.12727 30548469

[13] Deeg KH Rupprecht T. Pulsed doppler sonographic measurement of normal values for the flow velocities in the intracranial arteries of healthy newborns. *Pediatr Radiol.* (1989) 19:71–8. 10.1007/BF02387890 2646587

[14] Kluckow M Jeffery M Gill A Evans N. A randomised placebo-controlled trial of early treatment of the patent ductus arteriosus. *Arch Dis Child.* (2014) 99:F99–104. 10.1136/archdischild-2013-304695 24317704

[15] Ojala T Kaapa P Helenius H Ekblad U Kero P Valimaki I Low cerebral blood flow resistance in nonventilated preterm infants predicts poor neurologic outcome. *Pediatr Crit Care Med.* (2004) 5:264–8. 10.1097/01.PCC.0000112368.32965.45 15115565

[16] Perlman JM McMenamin JB Volpe JJ. Fluctuating cerebral blood-flow velocity in respiratory-distress syndrome. Relation to the development of intraventricular hemorrhage. *N Engl J Med.* (1983) 309:204–9. 10.1056/NEJM198307283090402 6866033

[17] Mosca F Bray M Lattanzio M Fumagalli M Tosetto C. Comparative evaluation of the effects of indomethacin and ibuprofen on cerebral perfusion and oxygenation in preterm infants with patent ductus arteriosus. *J Pediatr.* (1997) 131:549–54. 10.1016/S0022-3476(97)70060-X 9386657

[18] Arora R Ridha M Lee DS Elliott J Rosenberg HC Diop M Preservation of the metabolic rate of oxygen in preterm infants during indomethacin therapy for closure of the ductus arteriosus. *Pediatr Res.* (2013) 73:713–8. 10.1038/pr.2013.53 23493169

[19] Andersen CC Hodyl NA Ziegler NM Stark MJ. Determining the venous oxygen reservoir: a novel, hypothetical approach to titration of supplemental oxygen in preterm newborns. *Med Hypotheses.* (2018) 112:30–4. 10.1016/j.mehy.2018.01.002 29447932

[20] Schwarz CE Preusche A Wolf M Poets CF Franz AR. Prospective observational study on assessing the hemodynamic relevance of patent ductus arteriosus with frequency domain near-infrared spectroscopy. *BMC Pediatr.* (2018) 18:66. 10.1186/s12887-018-1054-6 29452581 PMC5816508

[21] Brown DW Hadway J Lee TY. Near-infrared spectroscopy measurement of oxygen extraction fraction and cerebral metabolic rate of oxygen in newborn piglets. *Pediatr Res.* (2003) 54:861–7. 10.1203/01.PDR.0000090928.93045.BE 12930911

[22] Schumann P Touzani O Young AR Verard L Morello R MacKenzie ET. Effects of indomethacin on cerebral blood flow and oxygen metabolism: a positron emission tomographic investigation in the anaesthetized baboon. *Neurosci Lett.* (1996) 220:137–41. 10.1016/S0304-3940(96)13210-9 8981492

[23] van der Hoeven MA Maertzdorf WJ Blanco CE. Relationship between mixed venous oxygen saturation and markers of tissue oxygenation in progressive hypoxic hypoxia and in isovolemic anemic hypoxia in 8- to 12-day-old piglets. *Crit Care Med.* (1999) 27:1885–92. 10.1097/00003246-199909000-00029 10507614

[24] Christmann V Liem KD Semmekrot BA van de Bor M. Changes in cerebral, renal and mesenteric blood flow velocity during continuous and bolus infusion of indomethacin. *Acta Paediatr.* (2002) 91:440–6. 10.1111/j.1651-2227.2002.tb01668.x 12061361

[25] Hammerman C Glaser J Schimmel MS Ferber B Kaplan M Eidelman AI. Continuous versus multiple rapid infusions of indomethacin: effects on cerebral blood flow velocity. *Pediatrics.* (1995) 95:244–8. 10.1542/peds.95.2.244 7838642

[26] FitzGerald GA Patrono C. The coxibs, selective inhibitors of cyclooxygenase-2. *N Engl J Med.* (2001) 345:433–42. 10.1056/NEJM200108093450607 11496855

[27] Forsey JT Elmasry OA Martin RP. Patent arterial duct. *Orphanet J Rare Dis.* (2009) 4:17. 10.1186/1750-1172-4-17 19591690 PMC2716300

[28] Orlando BJ Lucido MJ Malkowski MG. The structure of ibuprofen bound to cyclooxygenase-2. *J Struct Biol.* (2015) 189:62–6. 10.1016/j.jsb.2014.11.005 25463020 PMC4276492

[29] Beaudin AE Pun M Yang C Nicholl DD Steinback CD Slater DM Cyclooxygenases 1 and 2 differentially regulate blood pressure and cerebrovascular responses to acute and chronic intermittent hypoxia: implications for sleep apnea. *J Am Heart Assoc.* (2014) 3:e000875. 10.1161/JAHA.114.000875 24815497 PMC4309085

[30] Greisen G. Is near-infrared spectroscopy living up to its promises? *Semin Fetal Neonatal Med.* (2006) 11:498–502. 10.1016/j.siny.2006.07.010 16959556

[31] Wijbenga RG Lemmers PM van Bel F. Cerebral oxygenation during the first days of life in preterm and term neonates: differences between different brain regions. *Pediatr Res.* (2011) 70:389–94. 10.1203/PDR.0b013e31822a36db 21705960

[32] van der Laan ME Roofthooft MT Fries MW Berger RM Schat TE van Zoonen AG A hemodynamically significant patent ductus arteriosus does not affect cerebral or renal tissue oxygenation in preterm infants. *Neonatology.* (2016) 110:141–7. 10.1159/000445101 27088709

